# Integrated Multi-Omics Analysis Reveals the Role of the Gut Microbiota–Metabolite–Endocrine Axis in Post-Weaning Estrus Recovery in Tibetan Pigs

**DOI:** 10.3390/ani16111579

**Published:** 2026-05-22

**Authors:** Jian Zhang, Dong Yang, Mengjia Han, Mengqi Duan, Hongliang Zhang, Peng Shang

**Affiliations:** 1 College of Animal Science, Xizang Agriculture and Animal Husbandry University, Linzhi 860000, China; zhangjian@xza.edu.cn (J.Z.); 15086795164@163.com (D.Y.); 15515762043@163.com (M.H.); zduanduan0117@163.com (M.D.);; 2 Key Laboratory of Tibetan Pig Genetic Improvement and Reproduction Engineering, Linzhi 860000, China; 3 Tibetan Pig Science and Technology Courtyard in Linzhi, Linzhi 860000, China

**Keywords:** 16S rRNA sequencing, gut microbiota, HPG axis, metabolomics, reproductive hormones, Tibetan sows, weaning-to-estrus interval

## Abstract

The weaning-to-estrus interval (WEI) is an important indicator of sow reproductive performance, yet the mechanisms underlying post-weaning anestrus in Tibetan sows remain unclear. In this study, multiparous Tibetan sows were classified into an estrus group (FQ) and an anestrus group (WQ) based on estrus status after weaning. Serum reproductive hormones, hematological parameters, gut microbiota (16S rRNA sequencing), and fecal metabolites (untargeted metabolomics) were analyzed. Compared with the FQ group, the WQ group showed significantly lower estradiol (E_2_) and higher progesterone (P) levels (*p* < 0.01), along with a decreased proportion of neutrophils and an increased proportion of lymphocytes (*p* < 0.05). No significant differences in alpha diversity were observed, whereas PLS-DA revealed differences in microbial community structures between groups. LEfSe analysis indicated that *Methanobrevibacter* and *Acinetobacter* were enriched in the FQ group, whereas Muribaculaceae and *Prevotella* were enriched in the WQ group. Differential metabolites were mainly involved in amino acid and lipid metabolism and enriched in pathways related to steroid hormone biosynthesis, oocyte maturation, and tryptophan metabolism. These findings suggest that post-weaning anestrus may be associated with endocrine imbalances, immune changes, and gut microbiota–metabolite interactions. They may provide a basis for future studies in Tibetan pig breeding and genetic improvement.

## Simple Summary

Reproductive performance is a key factor affecting the efficiency of Tibetan pig production, and the weaning-to-estrus interval (WEI) is widely used to evaluate sow fertility. However, some Tibetan sows fail to return to estrus after weaning, which limits their reproductive performance, and the underlying causes remain unclear. In this study, we compared Tibetan sows that returned to estrus normally with those that remained in anestrus after weaning. By analyzing blood hormones, immune-related indicators, gut microbiota, and metabolites, we found that anestrus sows showed hormonal imbalance, altered immune status, and distinct changes in gut microbial composition and metabolic profiles. Further analysis suggested that gut microorganisms and their metabolites may regulate reproductive function by interacting with hormonal and metabolic pathways. These findings provide new insights into the biological mechanisms of post-weaning anestrus and may offer a basis for future studies on Tibetan pig breeding and genetic improvement.

## 1. Introduction

In the livestock production industry, the reproductive performance of sows is directly linked to breeding efficiency and overall industry development [1]. As a unique indigenous pig genetic resource in China, Tibetan sows are of considerable importance for studies on reproductive traits, particularly the regulatory mechanisms underlying estrus. Tibetan sows are mainly distributed across the Qinghai–Tibet Plateau and its surrounding regions, where they have long adapted to harsh environmental conditions characterized by high altitude, low temperature, hypoxia, and relatively limited feed resources, gradually developing distinctive biological characteristics [2]. Throughout this prolonged adaptive process, Tibetan pigs have also evolved specific estrus regulatory mechanisms and reproductive strategies [3].

During the reproductive process of sows, a relatively stable weaning-to-estrus interval (WEI, approximately 5–7 days) is critically important for subsequent ovulation, artificial insemination (AI), and embryo survival [4]. Previous studies have shown that the duration of WEI directly influences sow reproductive performance, including pregnancy rate and litter size [5]. Further studies have demonstrated that a prolonged WEI reduces pregnancy rate and embryo survival, whereas a shorter WEI is beneficial for improving embryo survival [6].

From a physiological perspective, WEI is primarily regulated by the positive and negative feedback mechanisms of reproductive hormones. Alterations in nutrient intake and metabolic status can lead to changes in metabolite levels as well as in the concentrations of related hormones (such as insulin, follicle-stimulating hormone (FSH), and luteinizing hormone (LH)), thereby affecting the function of the hypothalamic–pituitary–gonadal axis (HPG axis) and the estrous cycle [7]. Previous studies have shown that improving metabolic status during lactation not only enhances LH secretion but also increases the pulse frequency of FSH, promotes follicular development, and consequently shortens WEI [8,9]. In contrast, negative energy balance (NEB) has been reported to suppress HPG axis function, thereby contributing to lactational anestrus in sows [10].

The gut microbiota plays a key role in maintaining host digestive function, energy metabolism, and immune homeostasis [11]. A growing body of evidence indicates that the gut microbiota is closely associated with the physiological status of animals [12]. Previous studies in piglets have demonstrated substantial alterations in the alpha diversity, beta diversity, and overall community structure of the fecal microbiota during the weaning transition [13]. Similarly, during the estrous cycle of Tibetan sows, marked changes in physiological status may be accompanied by dynamic shifts in the gut microbial community structure [14]. Analysis of fecal samples from Tibetan sows using 16S rRNA sequencing may help characterize differences in gut microbiota structure and composition associated with estrus status, thereby helping elucidate mechanisms by which the gut microbiota may regulate estrus [15].

Therefore, this study takes Tibetan sows as the research object and systematically analyzes the differential characteristics and interactions of gut microbiota and metabolites between estrus and anestrus stages, aiming to provide a novel theoretical basis for elucidating the regulatory mechanisms underlying post-weaning anestrus in sows, and to provide a theoretical basis for improving reproductive performance in Tibetan sows.

## 2. Materials and Methods

### 2.1. Experimental Animals

The experimental animals and study design used in this study were approved by the Animal Protection and Use Ethics Committee of Tibet Agricultural and Animal Husbandry University (Approval No.: XZA-2005-012). A total of 80 multiparous, healthy Tibetan sows with similar body weights after weaning were selected as the research subjects. All experimental animals were housed at a pig farm located in Zengba Village, Nyingchi City, Tibet Autonomous Region, China. During the experimental period, standard immunization procedures and routine management practices of the farm were strictly followed, and a standardized feeding regimen was implemented to ensure free access to water.

### 2.2. Estrous Cycle Determination, Fecal and Blood Sample Collection

Eighty weaned Tibetan sows were subjected to batch weaning, and their estrus status was observed and recorded. Sows exhibiting estrus within 6–9 days after weaning were classified as the estrus group (FQ group), whereas those without estrus during this period were classified as the anestrus group (WQ group). Estrus detection was conducted twice daily (08:00 and 18:00) from day 1 to day 9 post-weaning. Estrus was identified based on behavioral and physiological indicators, including the standing reflex in response to back-pressure, vulvar swelling and redness, and increased activity, assisted by boar exposure. Fecal samples were collected from all sows in both groups on days 2, 5, and 8 post-weaning. To ensure comparability, fecal samples from the estrus (FQ) and anestrus (WQ) groups were collected on the same sampling days. For downstream analysis, a total of 18 fecal samples were randomly selected, including 9 samples from the FQ group and 9 samples from the WQ group. The selected fecal samples were immediately placed into sterile sampling tubes and rapidly frozen in liquid nitrogen. Peripheral blood samples were collected from five randomly selected sows per group on the second day after estrus onset in the FQ group. To ensure comparability between groups, blood samples from the WQ group were collected on the same day and at the same sampling time point as those from the FQ group. The relatively small sample size was mainly due to practical constraints associated with animal handling and experimental resources. In addition, similar sample sizes have been commonly used in exploratory studies investigating physiological and microbiome-related traits in pigs [13,15]. Therefore, the present study should be considered exploratory.

### 2.3. 16S rRNA Sequencing of Gut Microbiota

A total of 36 fecal samples were selected from the initial cohort of 80 sows, including 18 samples from the FQ group and 18 samples from the WQ group. The samples were selected based on consistent health status, similar parity, and the availability of complete experimental records, and were randomly chosen within each group for subsequent analyses. Genomic DNA was extracted from fecal samples of Tibetan sows using a DNA isolation kit, and the quality of the extracted DNA was assessed by the sequencing service provider using a Qubit 4.0 fluorometer (Thermo Fisher Scientific, Waltham, MA, USA). After genomic DNA extraction, the V3 + V4 regions of the 16S rDNA were amplified using specific primers with barcodes. The primer sequences were as follows: 341F: CCTACGGGNGGCWGCAG; 806R: GGACTACHVGGGTATCTAAT. The purified amplification products (amplicons) were used for sequencing library construction and subsequently sequenced on the Illumina PE250 platform (Illumina, San Diego, CA, USA).

### 2.4. Fecal LC–MS Metabolomics Analysis

Samples were vacuum freeze-dried and ground into powder using a grinding instrument (MM 400, Retsch, Haan, Germany,) at 30 Hz for 1.5 min. A total of 100 mg of powder was weighed and dissolved in 1.0 mL of extraction solution (methanol:water = 4:1, *v*/*v*), containing 0.02 mg/mL L-2-chlorophenylalanine as an internal standard. The dissolved samples were stored at 4 °C overnight, during which they were vortexed three times to improve extraction efficiency. After centrifugation (10,000× *g*, 10 min), the supernatant was collected, filtered through a microporous membrane (0.22 μm pore size), and then transferred into sample vials for LC–MS/MS analysis. The data acquisition system mainly consisted of an ultra-performance liquid chromatography system (Ultra Performance Liquid Chromatography, UPLC; Shimpack UFLC shimadzu CBM30A, (Shimadzu, Kyoto, Japan) and a tandem mass spectrometry system (MS/MS; Applied Biosystems 6500 QTRAP, Applied Biosystems, Foster City, CA, USA). An automatic sampler is maintained at 4 °C.

### 2.5. Blood Sample Analysis

Individual blood samples used for complete blood count (CBC) and white blood cell count and differential count (WCDC) were collected from the anterior vena cava into EDTA-containing anticoagulant tubes (disposable vacuum blood collection tubes, Tiannai Medical Devices, (Shanghai, China). Additional blood samples were collected into standard vacuum blood collection tubes (Huabo Medical Devices, Shanghai, China). All samples were transported in a refrigerated container at 4 °C and analyzed on the day of collection. Blood samples for reproductive hormone analysis were also collected from the anterior vena cava of sows. The samples were centrifuged at 4000 rpm for 15 min at 4 °C, and the plasma was separated and stored at −80 °C until further analysis.

## 3. Results

### 3.1. Blood Reproductive Hormones

Estradiol (E_2_) and progesterone (P) are both steroid hormones and represent key regulators of the estrous cycle. The levels of these hormones are commonly used to evaluate the WEI in sows. In this study, the serum reproductive hormone levels of the FQ group (estrus after weaning) were compared with those of sows showing anestrus after weaning (anestrus sows, WQ group), and the results are presented in Table 1.

Significant differences were observed between multiparous Tibetan sows that exhibited estrus after weaning and those that did not (*p* < 0.01). In contrast, the serum progesterone (P) concentration in the WQ group was significantly higher than that in the FQ group, also showing an extremely significant difference (*p* < 0.01). These results indicate that the secretion patterns of blood reproductive hormones in the Tibetan WQ group (anestrus after weaning) differ markedly from those in sows exhibiting normal estrus.

### 3.2. Blood Physiological Parameters

The results of the white blood cell differential count are presented in Table 2. Compared with the FQ group (estrus after weaning), the proportion of neutrophils in sows with post-weaning anestrus (WQ group) was significantly decreased (*p* = 0.027). In contrast, the proportion of lymphocytes in the WQ group was significantly higher than that in the FQ group (*p* = 0.034). No significant differences were observed between the two groups in the relative proportions of monocytes, eosinophils, or basophils (*p* > 0.05). In terms of absolute counts, no significant differences were detected between the two groups for neutrophils, lymphocytes, monocytes, eosinophils, or basophils (*p* > 0.05).

The results of the complete blood count parameters are shown in Table 3. No significant differences were observed between the estrus group (FQ) and the anestrus group (WQ) in related indices, including white blood cell count (WBC), red blood cell count (RBC), hematocrit (HCT), mean corpuscular volume (MCV), and mean corpuscular hemoglobin (MCH) (*p* > 0.05). Further analysis showed that the mean corpuscular hemoglobin concentration (MCHC) in the WQ group was significantly higher than that in the FQ group, with an extremely significant difference (*p* < 0.01).

### 3.3. Composition of Fecal Microbiota

Using high-throughput sequencing technology, 16S rRNA sequencing data from 36 fecal samples were analyzed at the OTU level. A total of 20 phyla, 28 classes, 63 orders, 106 families, 196 genera, and 160 species were identified. Among them, the FQ group contained 19 phyla, 28 classes, 63 orders, 102 families, 175 genera, and 132 species, whereas the WQ group contained 18 phyla, 25 classes, 56 orders, 94 families, 179 genera, and 128 species.

**Figure 1:**
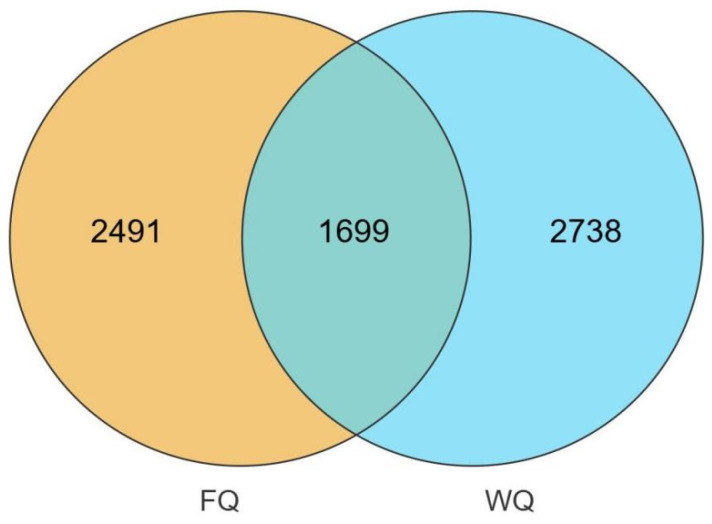
Venn diagram of species between the estrus group and the anestrus group.

Based on OTUs obtained at five taxonomic levels, a Venn diagram was constructed. As shown in Figure 1, a total of 6928 OTUs were identified in both groups, including 4190 OTUs in the FQ group and 4437 OTUs in the WQ group. The number of shared OTUs between the two groups was 1699, accounting for 24.52% of the total. In contrast, the number of unique OTUs in the FQ and WQ groups was 2491 and 2738, accounting for 35.95% and 39.52% of the total, respectively.

**Figure 2:**
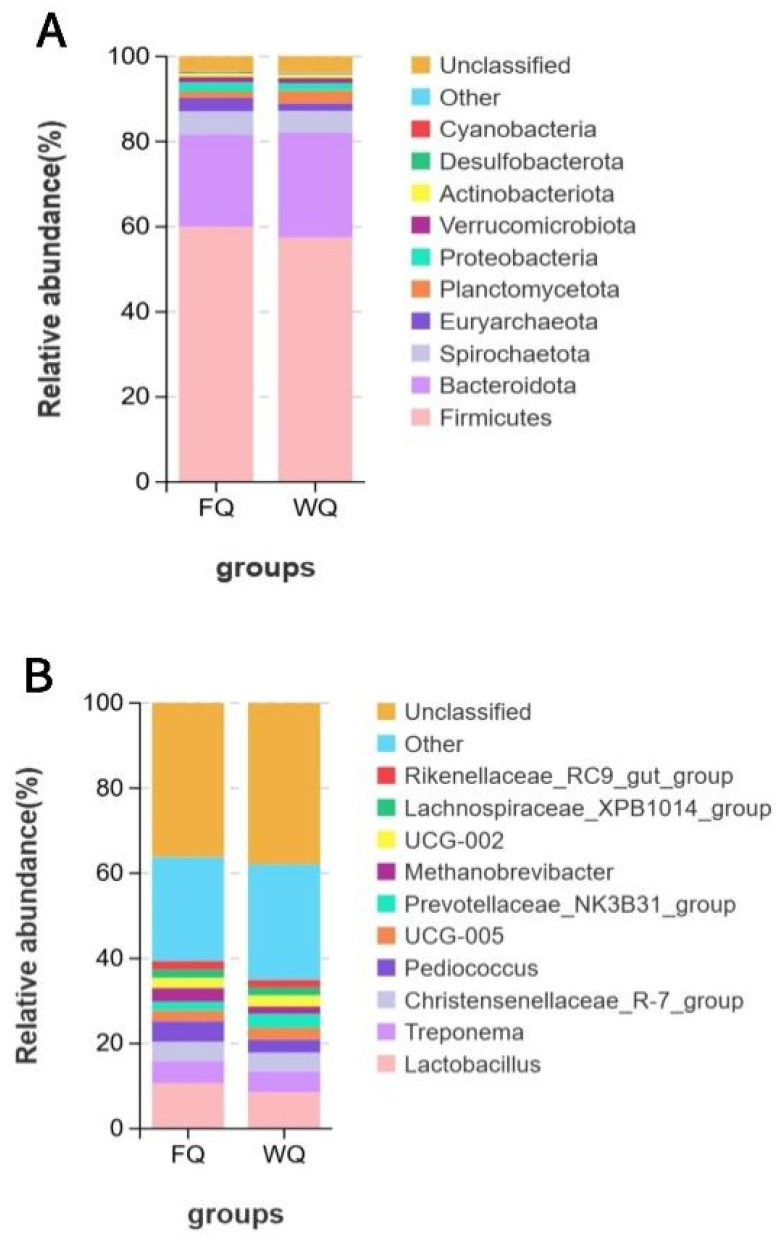
Analysis of microbial composition in the estrus and anestrus groups. (**A**) Stacked bar plot of species distribution at the phylum level; (**B**) stacked bar plot of species distribution at the genus level.

At the phylum level (Figure 2A), a total of 20 phyla were detected, and the overall composition of dominant phyla was largely consistent between the FQ and WQ groups. In both groups, Firmicutes and Bacteroidota were the dominant phyla. The relative abundances of Firmicutes were 59.9% and 57.0% in the FQ and WQ groups, respectively, whereas those of Bacteroidota were 21.8% and 24.7%, respectively. In addition, phyla such as Spirochaetota, Euryarchaeota, Proteobacteria, Actinobacteriota, and Planctomycetota were also detected, although their relative abundances were comparatively low.

At the genus level (Figure 2B), a total of 385 genera were annotated. *Lactobacillus* was the most abundant genus in both groups, with relative abundances of 10.6% and 8.5% in the FQ and WQ groups, respectively. This was followed by *Treponema*, with relative abundances of 5.3% and 5.1% in the two groups, respectively. In addition, the third most abundant genus in the FQ group was *Pediococcus*, with a relative abundance of 4.89%, whereas in the WQ group it was Christensenellaceae_R-7_group, with a relative abundance of 4.26%. The remaining genera were present at relatively low abundances and were classified as “Other” or unclassified taxa.

These results indicate that the overall fecal microbiota at both the phylum and genus levels was broadly similar between estrus and anestrus Tibetan sows after weaning; however, certain differences were observed in the relative abundances of specific dominant genera, providing a basis for subsequent differential microbial analysis and functional investigation.

### 3.4. Structural Characteristics of Fecal Microbiota and PLS-DA Discriminant Analysis

The results of alpha diversity analysis are presented in Table 4. No significant differences were observed between the estrus group (FQ) and the anestrus group (WQ) in the Sobs, Shannon, Simpson, Chao1, or ACE indices (*p* > 0.05), indicating comparable species richness and diversity levels between the two groups. Further principal coordinate analysis (PCoA) based on Bray–Curtis distances showed substantial overlap between the two groups in the two-dimensional ordination at the OTU level, without forming a clear separation. The first and second principal coordinates explained 13.52% and 9.35% of the community variation, respectively, suggesting that the overall structural differences in microbial communities between the two groups were limited.

**Figure 3:**
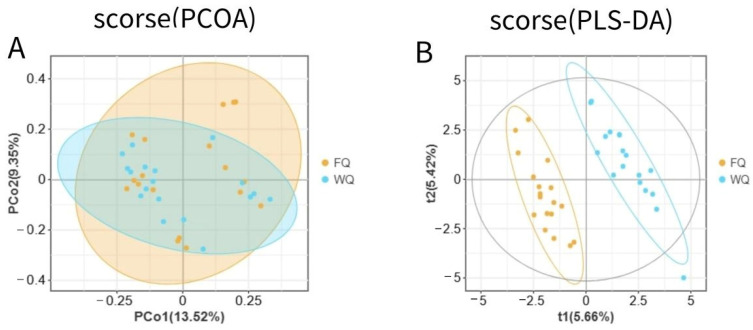
(**A**) Principal coordinate analysis (PCoA) score plot; (**B**) partial least squares discriminant analysis (PLS-DA) score plot.

However, in the partial least squares discriminant analysis (PLS-DA) (Figure 3B), samples from the estrus and anestrus groups exhibited a relatively clear grouping trend under the supervised model. Samples within each group were clustered together, and separation between groups was observed, indicating differences in microbial community composition between the FQ and WQ groups, with between-group variation exceeding within-group variation.

### 3.5. Differential Fecal Microbiota Between the Estrus and Anestrus Groups

**Figure 4:**
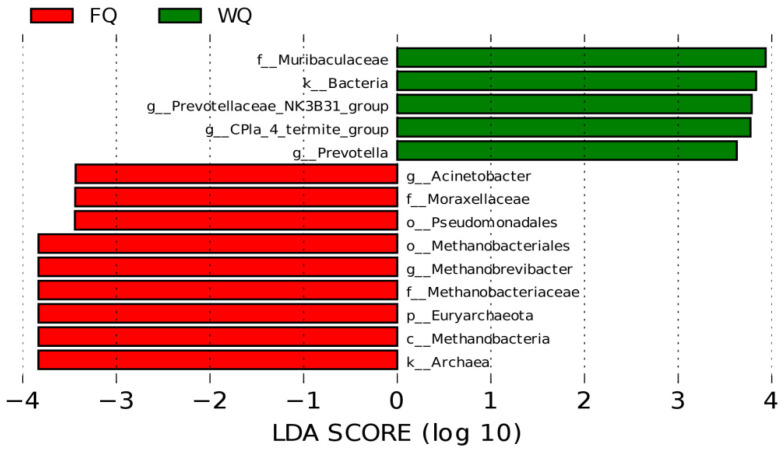
LEfSe analysis of differential taxa across multiple taxonomic levels.

To further identify differential taxa in the intestinal microbiota of Tibetan sows between the estrus group (FQ) and the anestrus group (WQ), linear discriminant analysis effect size (LEfSe) was applied to compare the two groups, with a screening threshold of LDA score ≥ 3.0. The results are shown in Figure 4.

The LEfSe analysis indicated that the relative abundances of gut microbial communities differed between the FQ and WQ groups, and 14 taxa were identified as having discriminative significance. Among them, nine differential taxa were enriched in the FQ group (Archaea, Methanobacteria, Euryarchaeota, Methanobacteriaceae, *Methanobrevibacter*, Methanobacteriales, Pseudomonadales, Moraxellaceae, and *Acinetobacter*). In contrast, five taxa were enriched in the WQ group (Muribaculaceae, Bacteria, Prevotellaceae, termite, and *Prevotella*).

In the FQ group, the taxon with the highest LDA score was Archaea, indicating that this taxon was significantly enriched in the gut of estrus sows. Further analysis at lower taxonomic levels revealed that Euryarchaeota, Methanobacteria, Methanobacteriales, Pseudomonadales, and Methanobacteriaceae showed increased relative abundances in the FQ group. In addition, among bacterial taxa, Moraxellaceae and its related taxa were significantly enriched in the FQ group. At the genus level, *Acinetobacter* and *Methanobrevibacter* exhibited significantly higher relative abundances in the FQ group compared with the WQ group (*p* < 0.05).

In contrast, multiple bacterial taxa were significantly enriched in the WQ group, among which Muribaculaceae showed the highest LDA score. At the domain level, the abundance of Bacteria was significantly increased in the WQ group. At the family level, Muribaculaceae exhibited a significantly higher relative abundance in the WQ group than in the FQ group (*p* < 0.05). At the genus level, Prevotellaceae_NK3B31_group, CPla_4_termite_group, and *Prevotella* were significantly enriched in the WQ group (*p* < 0.05).

### 3.6. Evaluation of Metabolomic Data Quality

To systematically evaluate differences in microbiota-derived metabolites between the estrus (FQ) and anestrus (WQ) groups of Tibetan sows, non-targeted metabolomics was applied to analyze fecal samples. A total of 19,253 metabolites were detected and identified (8560 in positive ion mode and 10,693 in negative ion mode), indicating a broad coverage of metabolites in this study.

**Figure 5:**
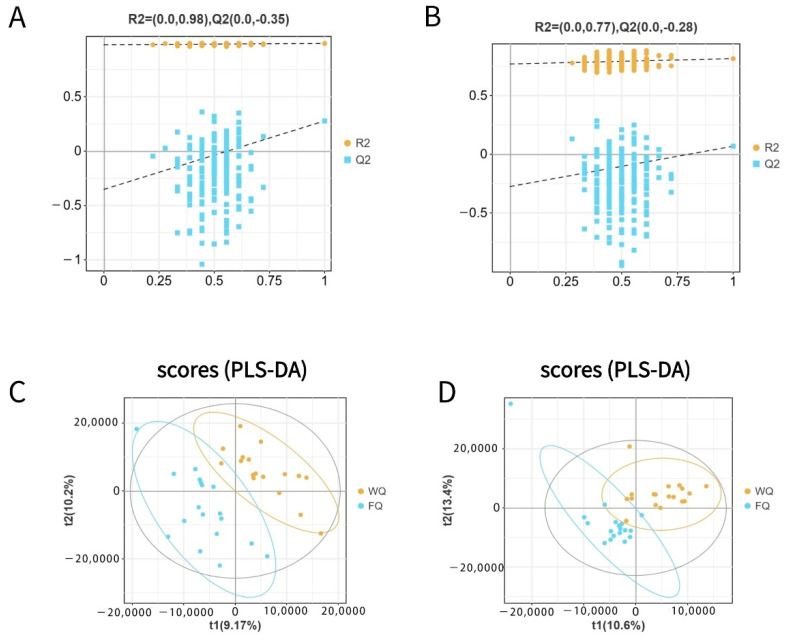
(**A**) OPLS-DA model (positive ion mode); (**B**) OPLS-DA model (negative ion mode); (**C**) PLS-DA score plot (negative ion mode); (**D**) PLS-DA score plot (positive ion mode).

PLS-DA and OPLS-DA models were constructed based on metabolite abundance data to assess group differences (Figure 5A–D). The PLS-DA results showed a clear separation between the FQ and WQ groups in the metabolic profiles, with good clustering within groups, suggesting differences in metabolite composition between the two groups (Figure 5C,D).

To validate the reliability of the model, permutation tests were performed for the OPLS-DA model. The PLS-DA results showed a clear separation between the FQ and WQ groups in the metabolic profiles, with good clustering within groups, suggesting differences in metabolite composition between the two groups (Figure 5C,D). Permutation tests were performed to validate the OPLS-DA model (Figure 5A,B). The results showed R^2^ values of 0.98 in positive ion mode and 0.77 in negative ion mode, and Q^2^ intercepts in both modes were ≤0, suggesting acceptable model stability and a limited risk of overfitting.

Based on the OPLS-DA model combined with an independent samples *t*-test (VIP ≥ 1, *p* < 0.05), a total of 263 significantly differential metabolites were identified. Among them, 57 differential metabolites were screened in the positive ion mode (15 upregulated and 42 downregulated), whereas 40 were identified in the negative ion mode (14 upregulated and 26 downregulated). Detailed information on all significantly differential metabolites is provided in Supplementary Table S1.

**Figure 6:**
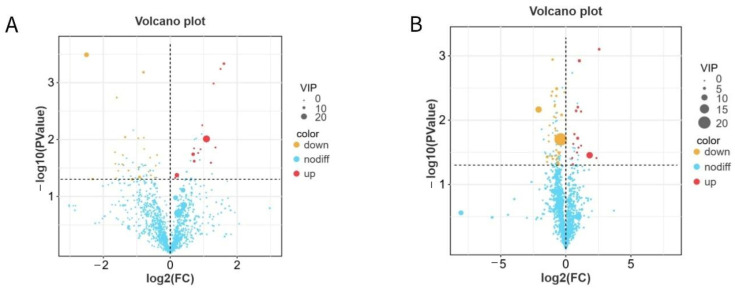
Volcano plots of differential metabolites between estrus and anestrus Tibetan sows. (**A**) Negative ion mode; (**B**) positive ion mode.

As shown in Figure 6A,B, the differential metabolites exhibited clear clustering patterns between the two groups, indicating that estrus status is closely associated with the composition of fecal microbiota-derived metabolites. Further analysis revealed that several key metabolites may be involved in metabolic and endocrine regulatory processes related to estrus. Among these, key discriminant metabolites—including 3-methyl-2-oxobutyric acid, succinic semialdehyde, and L-citrulline—are closely associated with amino acid metabolism, energy metabolism, and nitric oxide synthesis, and may influence ovarian function as well as local blood flow regulation. In addition, beta-glycerophosphate is involved in phospholipid metabolism and may affect the availability of precursors for steroid hormone synthesis, whereas erythritol is associated with oxidative stress and energy metabolism status. These alterations in metabolite profiles suggest that estrus may be accompanied by a remodeling of energy metabolism and endocrine regulatory networks.

**Figure 7:**
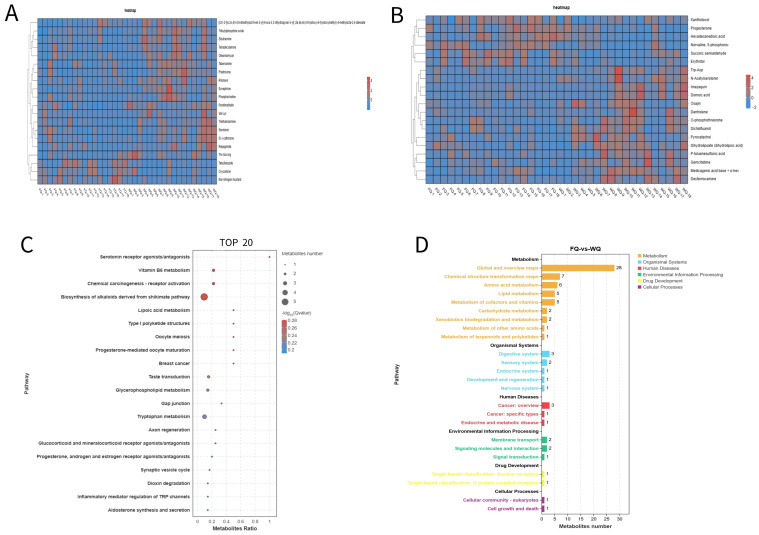
Clustering analysis of differential metabolites. (**A**) Positive ion mode; (**B**) negative ion mode; (**C**) KEGG pathway enrichment bubble plot; (**D**) KEGG pathway classification bar plot.

KEGG pathway enrichment analysis (Figure 7C) showed that the differential metabolites were mainly enriched in steroid hormone-related pathways (such as progesterone-mediated oocyte maturation), oocyte meiosis, tryptophan metabolism, and glycerophospholipid metabolism.

According to KEGG level 1 functional classification (Figure 7D), the enriched pathways were primarily associated with metabolic regulation, the endocrine system, and reproduction-related signaling pathways, suggesting that fecal microbiota-derived metabolites may participate in the regulation of estrus in Tibetan sows through coordinated multi-pathway interactions.

### 3.7. Correlation Analysis Between Fecal Microbiota and Metabolites

To explore the potential associations between fecal microbiota and metabolites, this study integrated differential microbial taxa obtained from 16S rRNA sequencing with differential metabolites identified by untargeted metabolomics, and Pearson correlation analysis was applied to evaluate their relationships [16]. Correlations with *p* < 0.05 were considered significant, and the correlation network was interpreted as an exploratory association analysis rather than evidence of causality. Multi-omics integrative analysis facilitates the elucidation of functional links between gut microbial composition and host metabolism, providing a basis for understanding microbe–host interaction mechanisms [17].

**Figure 8:**
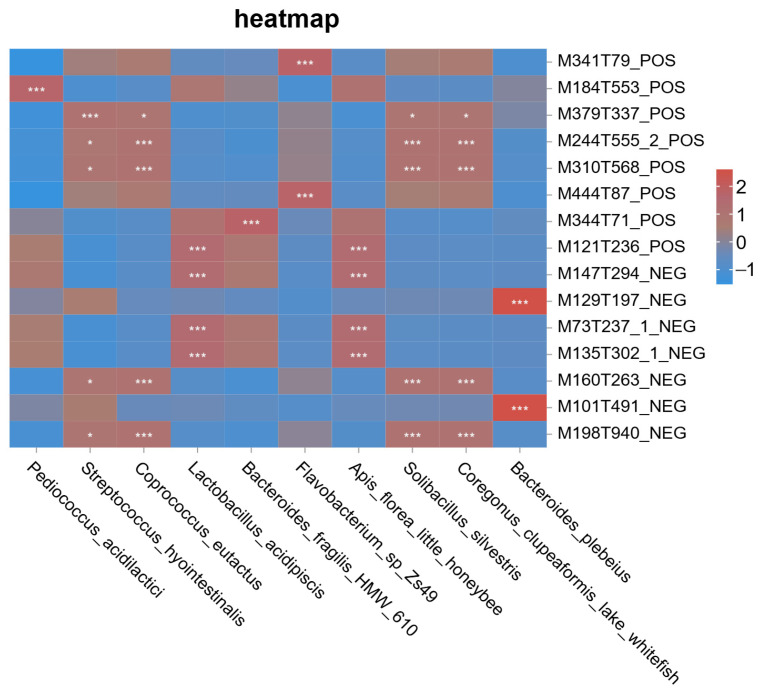
Correlation analysis between differential microbial taxa and differential metabolites. Pearson correlation coefficients are shown. * *p* < 0.05; *** *p* < 0.001.

As shown in Figure 8, multiple differential metabolites exhibited significant correlations with specific gut microbial taxa (*p* < 0.05 or *p* < 0.01), suggesting that certain microbial groups may be involved in the production or transformation of these metabolites. Among them, M310T568_POS (annotated as p-coumaraldehyde) showed a significant positive correlation with *Lactobacillus acidipiscis*. This phenolic metabolite is typically derived from the degradation of polyphenolic compounds by gut microbiota and possesses antioxidant and anti-inflammatory potential, which may contribute to the recovery of estrus by improving the local ovarian microenvironment. In addition, several amino acid-derived metabolites showed positive correlations with lactic acid bacteria and other commensal microbes, suggesting that gut microbiota may participate in energy supply and endocrine regulation through the modulation of amino acid metabolism.

## 4. Discussion

### 4.1. Endocrine–Hematophysiological Coordination in the Regulation of the Sow Estrous Cycle

Within the endocrine regulatory system dominated by the HPG axis, estradiol (E_2_) and progesterone (P), as key steroid hormones, play essential roles in follicular development, ovulation initiation, and the expression of estrous behavior [18]. Previous studies have demonstrated that increased E_2_ levels after weaning are closely associated with a shortened WEI, whereas abnormally elevated progesterone exerts negative feedback on the HPG axis, delaying follicular development and inhibiting the resumption of estrus [19,20,21].

The results of the present study showed that E_2_ levels in the FQ group were significantly higher than those in the WQ group, whereas P levels were significantly lower than those in the WQ group, further supporting the classical endocrine pattern in which “high E_2_–low P” favors rapid recovery of estrus after weaning [19,20].

In addition to endocrine changes, estrus recovery is accompanied by marked alterations in hematophysiological parameters. The significantly increased white blood cell (WBC) levels observed in the FQ group suggest enhanced immune activation during estrus, likely associated with follicular development and uterine tissue remodeling, which is consistent with the adaptive immune regulation reported during the estrous phase [22]. Meanwhile, the increased proportion of lymphocytes and decreased proportion of neutrophils in the WQ group indicate a shift in the immune system toward adaptive immunity under the “low E_2_–high P” condition [23].

Regarding erythrocyte parameters, increased MCV, MCH, and MCHC in the WQ group suggest that hormonal imbalance may influence erythropoiesis and hemoglobin metabolism, thereby altering red blood cell function [24,25]. In addition, hormonal dysregulation may further affect immune and metabolic status [26]. Taken together, differences in estrus status are not only reflected at the endocrine level but are also manifested as systemic physiological divergence through the immune and hematopoietic systems.

### 4.2. Role of Gut Microbiota and Metabolites in Estrus Regulation

In recent years, accumulating evidence has demonstrated a significant bidirectional interaction between gut microbiota and sex hormones [27,28,29]. In the present study, no significant differences were observed in α-diversity between the FQ and WQ groups; however, PLS-DA analysis showed a separation trend between the two groups, suggesting that estrus-related differences are primarily driven by changes in the abundance of specific functional taxa rather than alterations in the overall community structure. This finding is consistent with the relatively stable gut microbial architecture in pigs, which is typically dominated by Firmicutes and Bacteroidota [30].

Regarding differential taxa, *Methanobrevibacter* and *Acinetobacter* at the genus level, as well as Moraxellaceae at the family level, were enriched in the FQ group. Previous studies have shown that *methanogens* can enhance host energy utilization efficiency [31], *Acinetobacter* may regulate lipid metabolism via bile acid transformation [32], and Moraxellaceae is associated with immune modulation and may contribute to maintaining local immune homeostasis in the reproductive system [33]. Collectively, these taxa may be associated with follicular development and hormone synthesis.

In contrast, Muribaculaceae at the family level and *Prevotella* at the genus level were significantly enriched in the WQ group. Muribaculaceae has been reported to degrade the intestinal mucus barrier and induce chronic inflammation, thereby potentially disrupting the function of the hypothalamic–pituitary–gonadal (HPG) axis [34], whereas *Prevotella* is closely associated with lipid metabolism disorders and endocrine dysregulation [30]. In addition, *Prevotella* may participate in steroid metabolism and has been linked to variations in estrogen levels [35].

Metabolomic analysis revealed that differential metabolites were mainly enriched in pathways related to steroid hormone biosynthesis, oocyte maturation, and amino acid metabolism, suggesting that the gut microbiota may be associated with reproductive function through metabolic networks. SCFAs produced by Muribaculaceae may be involved in steroid hormone biosynthesis, potentially through pathways such as cAMP–PKA signaling [36,37], whereas certain metabolites (e.g., MAA) may inhibit estrogen receptor signaling, indicating a bidirectional regulatory effect [38].

At the level of specific metabolites, increased levels of erythritol and 3-methyl-2-oxobutyric acid were observed in the FQ group, reflecting enhanced energy and amino acid metabolism [39]. In contrast, metabolites such as L-citrulline and glycyrrhizin were closely associated with hormone regulation and follicular development [40,41,42]. KEGG pathway analysis further confirmed that differential metabolic pathways were mainly enriched in oocyte maturation and steroid hormone-related pathways [43].

### 4.3. Proposed Interaction Model of the Gut Microbiota–Metabolite–Endocrine Axis in Estrus Recovery

Based on the integrated analysis of gut microbiota, metabolites, and blood parameters in this study, a potential interaction model involving the gut microbiota–metabolite–endocrine axis in estrus recovery was proposed.

On the one hand, gut microbiota can produce a variety of bioactive molecules through metabolic processes, thereby participating in host physiological processes [44]. For example, this study identified a significant positive correlation between p-coumaraldehyde and *Lactobacillus acidipiscis*. This metabolite is derived from the degradation of dietary polyphenols [45] and can be further converted into p-coumaric acid by microbial activity, exerting antioxidant and anti-inflammatory effects [46]. Previous studies have shown that p-coumaric acid may influence the estrous cycle and reproductive organ development in female animals [47], while *Lactobacillus* species can inhibit inflammation by maintaining an acidic environment in the reproductive tract [48].

On the other hand, gut microbiota dysbiosis may affect the function of the hypothalamic–pituitary–gonadal (HPG) axis through the “gut–brain–reproductive axis”. The enrichment of Muribaculaceae in the WQ group may be associated with immune and endocrine changes via short-chain fatty acids [49,50], potentially involving estrogen signaling pathways through its metabolites [38]. Meanwhile, the increased abundance of *Prevotella* has been associated with polycystic ovary syndrome (PCOS) [51,52] and may disrupt hormonal balance through inflammation and insulin resistance [53,54,55]. In contrast, taxa enriched in the FQ group may be associated with energy metabolism, estrogen receptor activity, and inflammatory responses, thereby improving the responsiveness to estrogen [56,57,58,59]. However, the present study was observational and correlative in nature, and, therefore, it cannot determine whether endocrine dysfunction is a primary driver of gut microbial alterations or a downstream consequence of microbiota dysbiosis. Future studies should incorporate mechanistic validation approaches [15], including fecal microbiota transplantation (FMT), antibiotic-induced microbiota depletion, and controlled hormone intervention experiments, to distinguish microbiota-driven endocrine regulation from primary ovarian endocrine dysfunction [19,27]. In addition, germ-free or microbiota-controlled animal models combined with ovarian transcriptomic analysis may further clarify the causal relationships within the gut microbiota–metabolite–endocrine axis.

It should also be noted that Tibetan pigs are a unique high-altitude indigenous breed with a distinct genetic background, metabolic characteristics, and environmental adaptations [60,61]. Therefore, the gut microbiota–estrus associations identified in this study may not be fully generalizable to commercial pig breeds raised under different management and nutritional conditions. Future comparative studies involving commercial breeds are required to determine whether the observed microbiota–metabolite–endocrine interactions represent conserved reproductive regulatory mechanisms across pig populations.

### 4.4. Study Limitations

Several limitations of the present study should be acknowledged. First, the fecal metabolomics analysis reflects metabolite profiles in intestinal contents rather than systemic circulating metabolites or ovarian microenvironment metabolites [62]. Therefore, the detected metabolites may not directly represent ovarian metabolic status or endocrine activity. Second, although integrated correlation analysis suggested potential associations among gut microbiota, metabolites, and reproductive hormones, no direct evidence of microbiota–ovarian signaling pathways was obtained in this study [63]. In particular, ovarian tissue transcriptomics, receptor signaling analysis, and functional cellular experiments were not performed. Third, the relatively small sample size limits the statistical power of the study and may increase the risk of false-positive associations. Finally, because this study used a cross-sectional observational design, causal relationships between gut microbiota dysbiosis and endocrine dysfunction cannot be established. Future studies combining longitudinal sampling, microbiota intervention experiments, and ovarian functional validation are needed to clarify the mechanistic basis of post-weaning anestrus in Tibetan sows.

## 5. Conclusions

In summary, Tibetan sows with post-weaning anestrus exhibited distinct endocrine and hematological profiles compared with sows that returned to estrus after weaning, including lower estradiol, higher progesterone, and altered leukocyte composition. Although no significant differences were observed in alpha diversity, the gut microbial composition and fecal metabolite profiles differed between the two groups. Differential taxa and metabolites were associated with pathways related to steroid hormone biosynthesis, oocyte maturation, amino acid metabolism, and glycerophospholipid metabolism. These findings suggest a potential association among gut microbiota, metabolites, and estrus recovery in Tibetan sows and provide a foundation for future mechanistic studies aimed at improving reproductive performance.

## Figures and Tables

**Table 1:** Blood sex hormone concentrations in estrus and anestrus sows (Mean ± SEM).

	Estrus Sows	Anestrus Sows	*p*-Value
Estradiol (pg/mL)	66.292 ± 3.375 **	50.88 ± 3.051 **	*p* < 0.01
Progesterone (ng/mL)	41.02 ± 0.497 **	60.026 ± 3.48 **	*p* < 0.01

** There was a significant difference between the two groups of multiparous Tibetan sows that came into heat after weaning and those that did not come into heat after weaning (*p* < 0.01).

**Table 2:** White blood differential count parameters (relative values; mean + SD) of sows in estrus and sows not in estrus.

	Estrus Sows	Anestrus Sows
Neutrophils (%)	39.8 ± 2.972 *	28.925 ± 6.819 *
Lymphocytes (%)	48.767 ± 5.052 *	57.45 ± 6.418 *
Monocytes (%)	11.233 ± 1.25	12.275 ± 6.417
Eosinophils (%)	3.0667 ± 2.2189	5.125 ± 4.884
Basophils (%)	0.4667 ± 0.058	0.325 ± 0.05
Neutrophils (10^9^/L)	5.5925 ± 1.101	5.0833 ± 1.298
Lymphocytes (10^9^/L)	9.56 ± 0.665	10.828 ± 1.262
Monocytes (10^9^/L)	1.967 ± 0.571	2.17 ± 0.924
Eosinophils (10^9^/L)	0.4633 ± 0.291	0.588 ± 0.248
Basophils (10^9^/L)	0.077 ± 0.031	0.053 ± 0.005

* There was a significant difference between the two groups of multiparous Tibetan sows that came into heat after weaning and those that did not come into heat after weaning (*p* < 0.05).

**Table 3:** Complete blood count parameters (mean ± SD) of sows in estrus and sows not in estrus.

	Estrus Sows	Anestrus Sows
WBC (10^9^/L)	18.532 ± 4.523	17.2 ± 2.635
RBC (10^12^/L)	8.72 ± 0.483	8.186 ± 0.503
HCT (L/L)	49.925 ± 0.759	50.88 ± 2.565
MCV (FL)	57.375 ± 2.869	62.38 ± 4.679
MCH (pg)	18.275 ± 1.024	20.48 ± 1.199
MCHC (g/L)	318.75 ± 6.131 **	328.8 ± 8.786 **

** There was a significant difference between the two groups of multiparous Tibetan sows that came into heat after weaning and those that did not come into heat after weaning (*p* < 0.01).

**Table 4:** Alpha diversity indices of samples from different groups.

Sample Name	Estrus Sows	Anestrus Sows
Sobs index	3253.5 ± 67.24	3343.5 ± 72.27
shannon index	9.52 ± 0.34	9.81 ± 0.53
simpson index	0.989443 ± 0.007	0.992951 ± 0.005
Chao1 index	4003.67 ± 162.93	3817.00 ± 109.91
ACE index	3502.00 ± 110.99	3500.00 ± 64.57

## Data Availability

The data presented in this study are available from the corresponding author upon reasonable request.

## Supplementary Materials

The following supporting information can be downloaded at https://www.mdpi.com/article/10.3390/ani16111579/s1, Table S1. Differential metabolites in positive ion mode; Table S2. Differential metabolites identified in negative ion mode.
