# Supplementary material for: Integrated Multi-Omics Analysis Reveals the Role of the Gut Microbiota–Metabolite–Endocrine Axis in Post-Weaning Estrus Recovery in Tibetan Pigs
Source: Animals (Basel). 2026 May 22;16(11):1579. doi: 10.3390/ani16111579 (PMC13256035; doi:10.3390/ani16111579)
Supplement: Supplementary file 1 [file animals-16-01579-s001.zip › animals-4294184-supplementary.pdf]

Pvalue	VIP	MS2_name
0.0199	23.3759	N-butylamine
0.0351	9.4922	Benzalkonium chloride (c12)
0.0068	9.2851	L-citrulline
0.0012	5.2591	Diethylstilbestrol
0.0488	5.2084	Tabersonine
0.0191	5.2084	Desogestrel
0.0032	4.7278	Tributylphosphine oxide
0.0464	3.9989	Triethanolamine
0.0372	3.9045	Ethylmorphine
0.0083	3.8089	Myristamine oxide
0.0233	3.5722	Sibutramine
0.0063	3.4525	Tebufenozide
0.0072	3.0851	Nor-nitrogen mustard
0.0008	2.7413	7-methoxy-4-methylcoumarin
0.0011	2.6535	Arg-Lys
0.0042	2.5197	7-methyladenine
0.0363	2.4746	Danazol
0.0397	2.4701	(-)-secoisolariciresinol
0.0042	2.4031	Laurylguanidine
0.0466	2.2951	Serotonin
0.0400	2.2643	1,5-diaminonaphthalene
0.0165	2.2258	20-hydroxy-n-(2-hydroxyethyl)-5z,8z,11z,14z-eicosatetraenamide
0.0077	1.9994	Tetradecylamine
0.0036	1.9940	L-ng-monomethylarginine
0.0387	1.8638	Chlorambucil
0.0292	1.8515	Apiole
0.0249	1.8159	Harmine
0.0484	1.6715	Oxycodone
0.0319	1.6624	Melamine
0.0267	1.6296	Mifepristone
0.0057	1.5960	1-hexadecylamine
0.0447	1.5467	Prednisone
0.0073	1.5330	4,4-dimethyl-l-glutamic acid
0.0226	1.5324	Mescaline
0.0061	1.5304	4-acetyloxy-8-(3-oxo-2-pent-2-enylcyclopenten-1-yl)octanoic acid
0.0353	1.4050	Coniferin
0.0368	1.3927	S(-)-cathinone
0.0351	1.3872	(2-acetyloxy-3,6-diphenylcyclohexyl) acetate
0.0315	1.3836	Corticosterone 21-acetate
0.0215	1.3468	Phosphocholine
0.0446	1.3462	Val-Lys

0.0336	1.3212	[(2r)-2-[(e,2s,4r)-4,6-dimethyloct-6-en-2-yl]-6-oxo-2,3-dihydropyran-3-yl] (2e,4e,6s)-8-hydroxy-6-(hydroxymethyl)-4-methylocta-2,4-dienoate
0.0234	1.2641	Gelsemine
0.0134	1.2535	Menadione
0.0088	1.2369	12-hydroperoxy-5z,8z,10e,14z,17z-eicosapentaenoic acid
0.0120	1.2158	Oleandomycin
0.0388	1.2126	4-[(e)-3-hydroxy-8,10-dimethyl-2-(methylamino)dodec-6-enyl]phenol
0.0091	1.1991	9-nitrooleic acid
0.0195	1.1808	Ritonavir
0.0477	1.1767	Repaglinide
0.0316	1.1492	7.alpha.-hydroxydehydroepiandrosterone
0.0204	1.0630	D-erythro-sphinganine-1-phosphate
0.0104	1.0461	Gln-val
0.0488	1.0080	N-acetylneuraminate

Supplementary Table S1. Differential metabolites in positive ion mode

Pvalue	VIP	MS2_name
0.0097	23.693<br>6	Erythritol
0.0003	16.293<br>3	4-pyridoxic acid
0.0423	14.875<br>8	3-methyl-2-oxobutyric acid
0.0181	11.605<br>1	Succinic semialdehyde
0.0500	7.9467	P-toluenesulfonic acid
0.0240	7.8699	Progesterone
0.0454	7.7403	.beta.-glycerophosphate
0.0007	7.4241	3-furancarboxylic acid, tetrahydro-4-methylene-2-octyl-5-oxo-, (2r,3s)-
0.0005	6.9339	Norvaline, 5-phosphono-
0.0495	5.9644	D-glucarate
0.0091	4.2479	O-phosphothreonine
0.0256	4.0903	3-dehydrocholic acid
0.0010	3.2527	3-hydroxyanthranilic acid
0.0006	2.8983	4-aminosalicylic acid
0.0018	2.7099	N-Acetylserotonin
0.0473	2.6808	Xanthotoxol
0.0138	2.4049	20-hydroxy-4z,7z,10z,13z,16z,18e-docosahexaenoic acid
0.0272	2.3415	7-hydroxyflavanone
0.0351	2.1721	Celastrol
0.0310	2.0700	Bisdemethoxycurcumin
0.0056	1.9905	4-hydroxy-3-methoxybenzyl alcohol
0.0424	1.9518	2-amino-3-[hydroxy-[2-hydroxy-3-[octadec-9-enoyl]oxypropoxy]phosphoryl]oxypropanoic acid
0.0172	1.8265	Hexadecanedioic acid
0.0467	1.7611	Dihydrolipoate (dihydrolipoic acid)
0.0148	1.7449	Cycloserine
0.0095	1.6220	Desferrioxamine
0.0477	1.5605	Dantrolene
0.0187	1.4385	2-keto-l-gulonic acid
0.0143	1.4328	4-nitrocatechol
0.0188	1.4009	Domoic acid
0.0288	1.3743	Gemcitabine
0.0209	1.3282	Medicagenic acid base + o-hex
0.0146	1.1538	Osajin
0.0266	1.1236	Trp-Asp
0.0480	1.1055	Benzoic acid, 3,6-dichloro-2-methoxy-

0.0354	1.0931	Cylindrospermopsin
0.0366	1.0866	DL-asparagine
0.0284	1.0630	Pyrocatechol
0.0167	1.0499	Imazaquin
0.0093	1.0489	Dichlofluanid

Supplementary Table S2. Differential metabolites identified in negative ion mode
